# Isolation and characterization of two novel halotolerant Catechol 2, 3-dioxygenases from a halophilic bacterial consortium

**DOI:** 10.1038/srep17603

**Published:** 2015-12-01

**Authors:** Guang Guo, Tingting Fang, Chongyang Wang, Yong Huang, Fang Tian, Qijia Cui, Hui Wang

**Affiliations:** 1 State Key Joint Laboratory of Environment Simulation and Pollution Control, School of Environment, Tsinghua University, Beijing, 100084, China

## Abstract

Study of enzymes in halophiles will help to understand the mechanism of aromatic hydrocarbons degradation in saline environment. In this study, two novel catechol 2,3-dioxygenases (C23O1 and C23O2) were cloned and overexpressed from a halophilic bacterial consortium enriched from an oil-contaminated saline soil. Phylogenetic analysis indicated that the novel C23Os and their relatives formed a new branch in subfamily I.2.A of extradiol dioxygenases and the sequence differences were further analyzed by amino acid sequence alignment. Two enzymes with the halotolerant feature were active over a range of 0–30% salinity and they performed more stable at high salinity than in the absence of salt. Surface electrostatic potential and amino acids composition calculation suggested high acidic residues content, accounting for their tolerance to high salinity. Moreover, two enzymes were further characterized. The enzymes activity both increased in the presence of Fe^3+^, Fe^2+^, Cu^2+^ and Al^3+^ and showed no significant inhibition by other tested metal ions. The optimal temperatures for the C23Os were 40 °C and 60 °C and their best substrates were catechol and 4-methylcatechol respectively. As the firstly isolated and characterized catechol dioxygenases from halophiles, the two halotolerant C23Os presented novel characteristics suggesting their potential application in aromatic hydrocarbons biodegradation.

The saline and hypersaline environments, such as oil fields, marine habitats, salt marshes, natural saline lakes and saline industrial effluents, are subjected to contamination with high levels of petroleum hydrocarbons^1^,^2^. Contamination of these ecosystems constitutes a serious environmental problem due to the high toxicity exhibited by the aromatic hydrocarbons, which belong to a class of environmentally persistent compounds^3^. Microbial degradation has been deemed as the most effective and important strategy for aromatic hydrocarbons elimination. Much research has been done on elucidating the ability of microorganisms to degrade aromatic hydrocarbons in terrestrial and marine environments^2^,^4^,^5^. However, conventional microorganisms are unable to degrade organic compounds efficiently in saline conditions^6^. One alternative to solve this problem is the use of halophilic microorganisms adapted to these conditions. Several studies have demonstrated that halophilic bacteria grow well and can degrade aromatic hydrocarbons effectively in saline or hypersaline environments^6^,^7^. These reports suggested that halophilic microorganisms possess great potential in bioremediation of saline environments contaminated by aromatic hydrocarbons.

Biodegradation of aromatic hydrocarbons in saline and hypersaline environments has attracted strong attention in recent years. Much research focused on ecological studies^6^,^8^,^9^, strain identification^3^,^10^,^11^,^12^ and various hydrocarbons utilization^1^,^13^,^14^,^15^ in saline environments. However, only a few studies are available concerning the genes and enzymes involved in aromatic hydrocarbons degradation in halophiles. In the degradation pathways of aromatic hydrocarbons by non-halophiles, aromatic ring cleavage played a central role in the complete mineralisation of these compounds. The scission of the aromatic ring was catalyzed by different types of dioxygenases, such as catechol 1, 2-dioxygenase (C12O), catechol 2, 3-dioxygenase (C23O), protocatechuate 3, 4-dioxygenase, protocatechuate 4, 5-dioxygenase, and the cleavage occurred at *ortho*- or *meta*-position under aerobic conditions^3^,^16^,^17^. As for halophilic bacteria, Garcia *et al.*^3^ have showed the presence of the genes encoding catechol 1, 2-dioxygenase and protocatechuate 3, 4-dioxygenase involved in aromatic compounds degradation. Two gentisate 1, 2-dioxygenase encoding genes were also found in halophilic archaea which degraded benzoate^18^,^19^. Moreover, Kim *et al.*^20^ and Moreno *et al.*^21^ futher separately studied the genetic organization of benzoate and benzene-degrading gene clusters from two halophiles to elucidate the compounds degradation pathway. Overall, these studies suggested that halophilic bacteria might employ the metabolic pathways similar to those in non-halophilic bacteria. Despite of this, deeper degradation regulation mechanisms by halophilies are still unknown, for example, the specific properties of enzymes during degradation process. Therefore, in-depth studies are still needed to obtain greater insights into the enzymes involved in aromatic hydrocarbons degradation in saline environments.

As a crucial enzyme involved in the degradation of aromatic hydrocarbons, catechol 2, 3-dioxygenase (C23O, key enzyme of *meta*-cleavage pathway) has been identified as one of the most widespread ring cleavage enzymes^22^ and many C23Os have been reported from non-halophilic bacteria (*Pseudomonas*, *Sphingomonas*, *Acinetobacter*, *Bacillus*, *Ralstonia*, *Burkholderia* and *Nocardia*)^23^. As a member of extradiol dioxygenases, C23Os from non-halophiles had a well characterized phylogeny based on their amino acid sequences and could be divided into several subfamilies and families^24^,^25^. However, the information about the C23Os from halophilic or halotolerant bateria is scarce. To our best knowledge, there are only a few reports revealed the presence of genes encoding catechol 2, 3-dioxygenase in halophilic microbial cultures by polymerase chain reaction (PCR) detection^2^,^9^,^16^,^26^ or by draft genome sequence^27^, and no further in-depth research could be obtained on the catechol 2, 3-dioxygenase genes and enzymes in halophiles. Searching more C23Os from various bacterial sources in diverse surroundings will help in deeply understanding this kind of enzyme and promoting its efficiency in degradation application. So far, purified or characterized C23Os of halophile origin have not yet been studied and current knowledge of their genetic characteristics, biochemical properties, substrate specificity, and their halo-stability properties is also lacking.

In this work, we firstly cloned, expressed and purified two novel C23Os from a moderately halophilic bacterial consortium. Then a description about the newly found enzymes in the C23O subfamily was provided based on phylogenetic analysis and amino acid sequence alignment. Furthermore, some application properties of these two enzymes were characterized. We particularly investigated the effects of salts on enzyme activity and stabilities. This study helps to develop the knowledge of the role of halotolerant enzymes in aromatic hydrocarbons degradation by halophilic bacteria and to understand the specific properties presented by halotolerant catechol dioxygenases for potential application.

## Results

### Phenanthrene-degrading bacterial consortium description and C23O enzyme assay

In the present work, a halophilic bacterial consortium (HF-1), which showed considerable growth with yellowish color indicating ring cleavage of the aromatic compounds, was enriched from a saline soil using phenanthrene as the sole carbon and energy source at 10% salinity. HF-1 could efficiently degrade phenanthrene (100 mg/L) at as high as 20% (w/v) NaCl concentration within 4 days (Fig. S1). The microbial community structure at genus level was studied by a pyrosequencing analysis based on the bacterial 16S rRNA gene. A wide range of bacterial groups were identified as *Thalassospira*, *Nitratireductor*, *Fodinicurvata*, *Tistlia*, *Rhodobium*, *Marispirillum*, *Pelagibius*, *Amorphus*, *Mariprofundus*, *Alcanivorax*, *Marinobacter*, *Chromohalobacter* and *Psychroflexus* (data not shown).

A dynamic pattern of C23O activity of this bacterial consortium was measured during degradation of phenanthrene at 10% salinity (Fig. S1). During the period of phenanthrene degradation, the C23O activity increased slowly from 3.8 U at the initial phase, then advanced rapidly and achieved a peak value of 36.1 U when phenanthrene was depleted completely. In addition, C23O activity could remain at a high level even after the added substrate was consumed. As C23O was mainly induced by catechol, one of the intermediates during phenanthrene degradation, we believe the remaining activity could be due to the presence of high levels of catechol when phenanthrene was completely removed from the system (catechol was not measured). This phenomenon suggested that C23O played a part in this phenanthrene mineralization process.

### Clone library screening and expression profile of C23O genes

To obtain C23O genes present in the halophilic bacterial consortium, a clone library was constructed. Two novel C23O genes (924 bp), named C23O1 gene and C23O2 gene respectively, were found in the clone library. The similar result was also observed by PCR-DGGE analysis of C23O genes in HF-1. These two fragments showed 82% DNA sequence similarity and 90% amino acid identity with each other.

**Figure 1:**
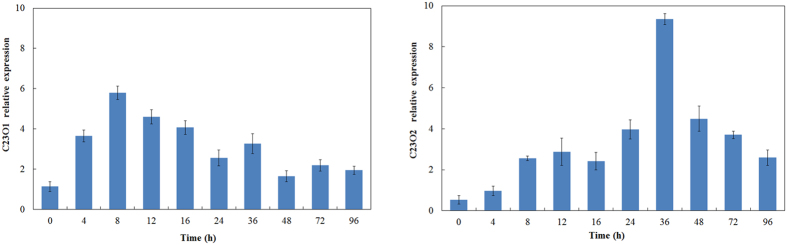
Relative expression levels of the C23O genes during phenanthrene degradation. The transcript/gene ratios were calculated for the microcosm incubation. Three independent experiments were performed. Vertical bars represent the mean of three measure replicates and three microcosm replicates (i.e. mean of nine measures).

To examine whether the two C23O genes are specifically involved in phenanthrene degradation process, expression profile of the C23O genes was measured by RT-PCR. The results revealed the expression of both genes during phenanthrene degradation process (Fig. 1). C23O1 was expressed rapidly after phenanthrene addition. The relative expression of C23O1 reached a peak value at 8 h, while that of C23O2 reached the peak value at 36 h. Although the expression of C23O2 subsequently decreased after 36 h, the relative expression level still remained higher than that in the beginning. The two C23O genes showed different expression profile in this phenanthrene degradation process.

### Overexpression and purification of C23Os

**Figure 2:**
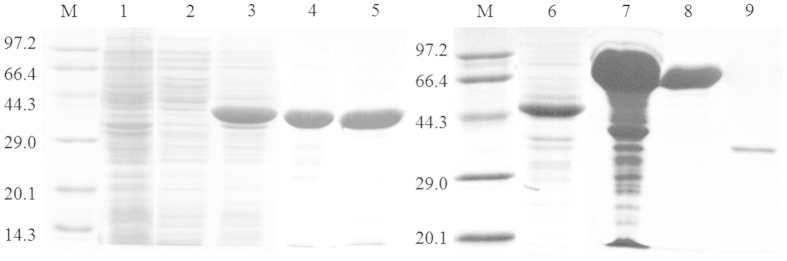
Detection of C23O1 and C23O2 overproduced in pET28a/C23O1 and pMAL-c5X/C23O2. High amounts of 35 kDa were mainly soluble. M: maker;1: pET28a in the precipitation (major amount of insoluble protein); 2: pET28a in supernatant (minor amount of protein); 3: pET28a/C23O1; 4: purified pET28a/C23O1; 5: purified C23O1 after digested; 6: pMAL-c5X; 7: pMAL-c5X/C23O2; 8: purified pMAL-c5X/C23O2; 9: purified C23O2 after digested.

The C23O1 and C23O2 genes were subcloned into pET-28a (+) and pMAL-c5X respectively, and overexpressed in *E.coli* BL21 (DE3) with 0.5 mM IPTG induction. The recombinant C23Os were purified by chromatography and the purification process was analyzed by SDS-PAGE (Fig. 2). The results indicated that both proteins were correctly synthesized into *E. coli* host cells and the molecular mass of the two purified proteins were approximately 35 kDa. After digestion with protease, the two purified enzyme showed the activity values up to 136.7 U and 52.3 U respectively. No C23O activity was detected in the control strain BL21 (ED3)-pET-28a (+) and BL21 (ED3)-pMAL-c5X.

### Taxonomic identification of C23Os

**Figure 3:**
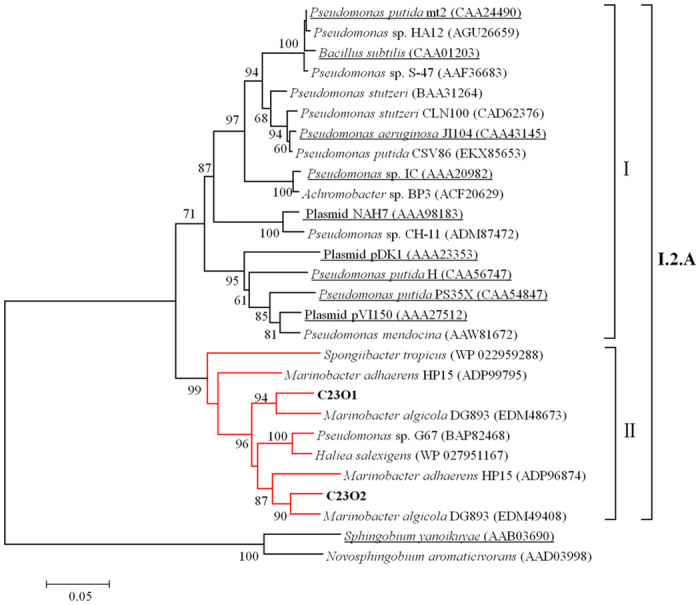
Phylogenetic neighbor-joining tree based on the C23O amino acid sequences. The clones isolated in this study are indicated with boldface type. The sequences with the underline are previously reported C23Os from non-halophiles (classic C23Os) which were classified by Eltis and Bolin^24^. The C23O amino acid sequences of *Sphingobium yanoikuyae* (AAB03690) and *Novosphingobium aromaticivorans* (AAD03998), two members of subfamily I.2.B were used as an outgroup. Bootstrap values (>60) expressed as percentages of 1000 replications are indicated at the branch points. Bar, 0.05 substitutions amino acid per site.

BLASTp analyses revealed that the two C23Os showed highest similarity (95% and 93%) with the proteins from *Marinobacter algicola* DG893. The sequence similarities with other C23Os were summarized in Table S1. Two phylogenetic trees were constructed with deduced amino acid sequences of C23O enzymes selected from subfamily I.2.A (Fig. 3 and Fig. S2). Phylogenetic neighbor-joining tree analysis (Fig. 3) presented that C23O1, C23O2 and their seven relatives formed a unique branch (group II) in the tree, and they cannot be confidently assigned to the branch with the previously reported C23Os from non-halophiles (classic C23Os) classified by Eltis and Bolin^24^ (group I). The maximum-parsimony tree presented the same results (Fig. S2). The two phylogenetic trees showed the new formed branch with high bootstrap support: 99% with the neighbour-joining method (Fig. 3) and 93% with the maximum-parsimony method (Fig. S2), respectively. In group II, C23O1 and C23O2 showed highest homology with putative protein EDM48673 and EDM49408, two proteins only described from whole-genome sequences of the halophilic bacteria *Marinobacter algicola* DG893^28^. Another four relatives are also the proteins predicted from whole-genome sequences from *Marinobacter adhaerens* HP15, *Haliea salexigens* and *Spongiibacter tropicus*.

### Amino acid sequence analysis of C23Os

To further illustrate that the two novel C23Os were classified as members in subfamily I.2.A, amino acid sequences alignment between the novel and classic C23Os (mentioned in Fig. 3) were performed. The analysis exhibited that the conserved domains, including Fe-ligands (His153, His214, and Glu265), four substrate-binding sites as well as twelve residues forming the inner channel wall of the active site pocket, were all consistent in the aligned sequences as highlighted in Fig. S3. Especiallly the eight evolutionally conserved active sites (His153, Ala 189, Phe 191, His 199, His214, His 246, Tyr 255 and Glu265) of the novel C23Os were identical to those classic ones, one of which had been previously structurally studied^29^, validating that the two novel enzymes could be assigned to subfamily I.2.A. On the other hand, comparison of the amino acid sequences allowed identification of specific residues important for enzyme distinction in the two C23Os (Fig. S3). Some obvious residue conversions could be noticed in novel C23Os compared with those classic ones: a number of negative residues-related changes and several positive residues-related changes; In addition, there also existed several hydrophobic and polar residues shifts (yellow backcolor in Fig. S3).

### Amino acids composition calculation

Table 1 calculated the amino acids composition of the selected C23Os to summarize the residue differences, presenting the content of the charged amino acids, hydrophobic and polar residues in C23Os. The information showed that the positive residues ratio were consistent between novel and classic C23Os, while the negative residues content were higher in novel C23Os compared with classic ones. Meanwhile, C23O1 possessed more negative residues than C23O2. In addition, the novel C23Os had a lower polar residues content than those classic ones.

### Electrostatic potential analysis of C23Os

Electrostatic potential provides information about the net electrostatic effect produced by total charge distribution (electron + proton)^30^ and negative electrostatic potential is usually consistent with acidic amino acids content on the enzyme surface. The electrostatic potential of the two C23Os was estimated and the distribution of charges was displayed in Fig. S4. C23O1 and C23O2 showed higher negative electrostatic potential on the surface compared with C23O from *Pseudomonas putida* mt2, a classic protein that had been well structurally studied. More specifically, C23O1 presented a higher negative potential from the right and the top view while C23O2 exhibited a higher one on the front of the structures. On the other hand, in the analysis of some residue conversions in Fig. S3, it could be noticed that the charged (negative and positive) residue-related changes were mostly located on the surface (marked with **s**), supporting the above results of the surface electrostatic potential changes.

### Effect of salinity on C23Os activity and stability

**Figure 4:**
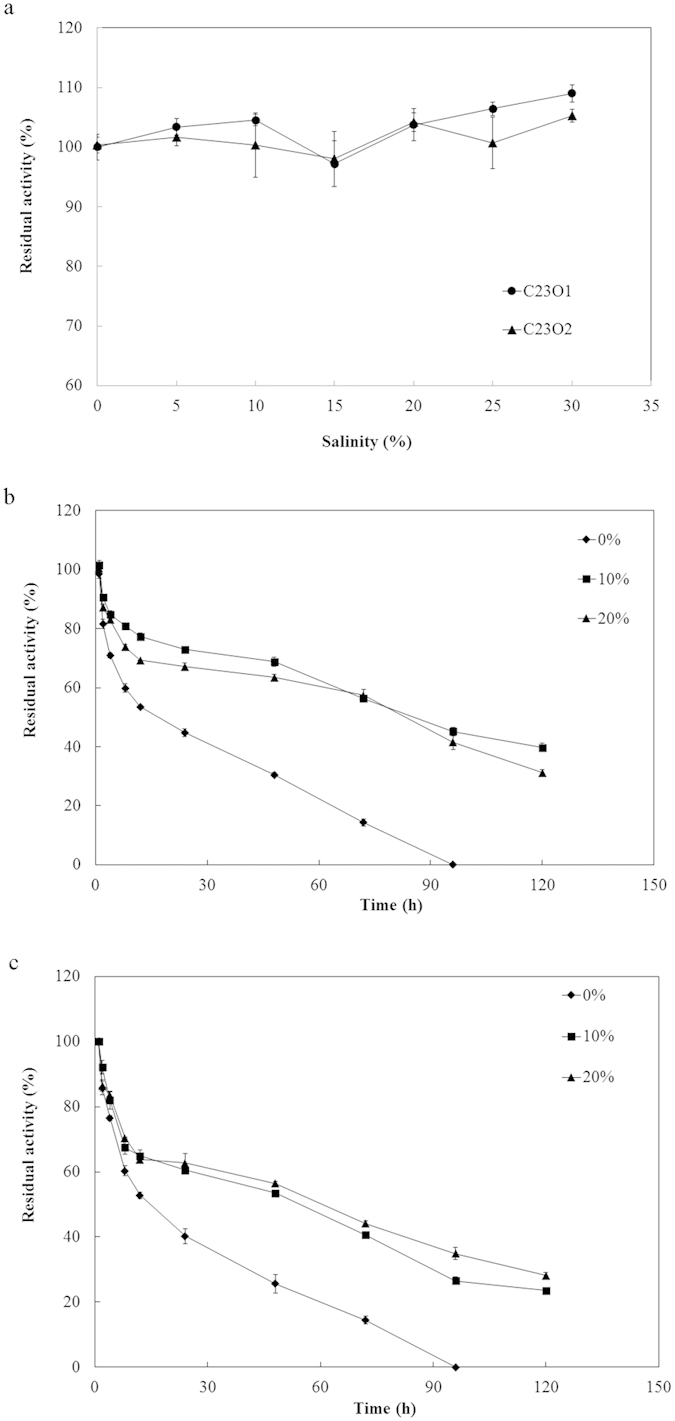
Effects of NaCl concentration on enzyme activity (**a**), and halotolerance of C23O1 (**b**) and C23O2 (**c**) after a pre-incubation of indicated time. Aliquots of the enzyme at different time points were taken and the residual activity was measured according standard assay procedure. The value obtained without NaCl in the reaction was taken as 100%. The values shown represent averages from triplicate experiments. Error bars represent the standard deviation.

To determine the effect of salinity on the enzyme activity and stability, a test using gradient salt concentrations ranging from 0 to 30% was performed. The enzyme activity examination (Fig. 4a) revealed that the two C23Os were both highly active over a broad range of NaCl concentration, even as high as 30% salinity, while C23O1 presented a little higher activity than C23O2. Particularly, two enzymes kept high activity at 0% NaCl concentration, suggesting C23O1 and C23O2 the halotolerant feature instead of halophilic character. The results of time-dependent enzyme stability (Fig. 4b,c) demonstrated enzyme activity could be more stable at 10% and 20% NaCl concentration than in the absence of NaCl. Residual activities of C23O1 and C23O2 were 77.3% and 69.3% at 10% NaCl (24 h) and 60.5% and 62.7% at 20% NaCl (24 h), while the absence of salt made the C23O1 and C23O2 activity reduce to below 60% after 24 h and lose activity at 96 h.

### Effect of temperature on C23Os activity

**Figure 5:**
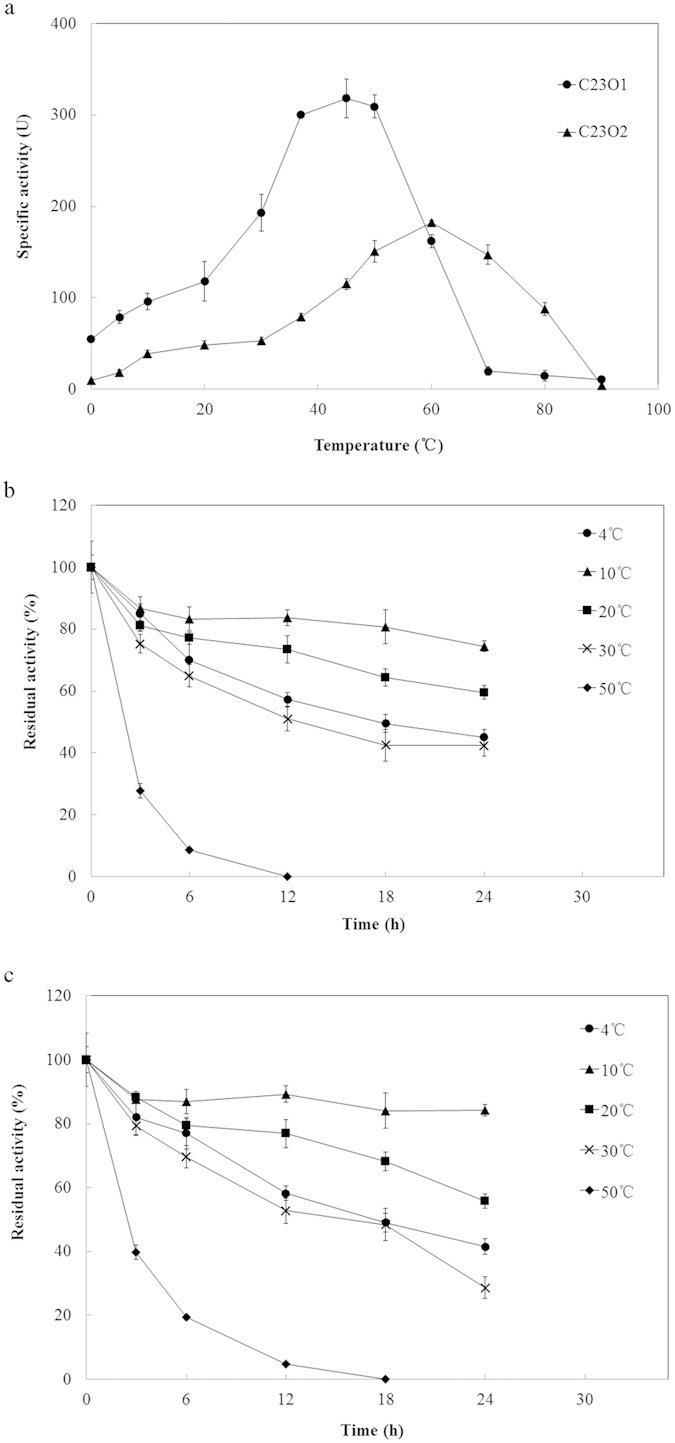
Effect of temperature on C23O activity (**a**), and thermal stability of C23O1 (**b**) and C23O2 (**c**). The values shown represent averages from triplicate experiments. Error bars represent the standard deviation.

The influence of reaction temperature on enzyme activity was investigated at the temperatures range of 0–90 °C using catechol as substrate. The two enzymes showed different optimal temperature: C23O1 demonstrated a high optimal temperature of 40 °C, while C23O2 required a temperature of 60 °C to exhibit the maximal activity (Fig. 5a). What’s more, the specific activity of C23O1 was 1.9 times as much as that of C23O2 at the optimal temperature. In addition, the C23O2 activity could still be detected at 80 °C with 54% of activity at the optimal temperature. In the test of temperature effect on time-dependent activity (Fig. 5b,c), the results showed the similiar trends between the two C23Os that they were both highly stable in the temperature range of 4–30 °C for 24 h. Their activities were kept best at 10 °C and the residual activities were about 80% at 24 h, followed by 20 °C with approximately 60% residual activies at 24 h. Their enzyme activities declined rapidly as the temperature increased above 50 °C.

### Effect of metal ions on C23Os activity

The effect of different metal ions on enzyme activity was investigated, using catechol as a substrate (Table 2). The results revealed that most of metal ions increased the enzyme activity. C23O1 and C23O2 activities were both enhanced by Fe^3+^, Fe^2+^, Cu^2+^ and Al^3+^ and the effects of Fe^2+^ and Fe^3+^ were higher than other metal ions. C23O1 exhibited the highest enzyme activity (265.65%) with Fe^2+^ added, while C23O2 showed the largest activity (377.37%) in the presence of Fe^3+^. On the other hand, Mn^2+^ did not significantly affect the two C23Os activity. However, C23O1 activity was slightly inhibited by K^+^ and Mg^2+^, which did not influence the C23O2 activity obviously.

### Substrate specificity of C23Os

The relative C23O activities toward various catecholic compounds (3-methylcatechol, 4-methylcatechol, 4-chlorocatechol and 1, 2-dihydroxynaphthalene) were examined (Table 3). C23O1 and C23O2 showed activity against 3-methylcatechol, 4-methylcatechol and 4-chlorocatechol, but could not oxidize 1, 2-dihydroxynaphthalene. The C23O1 presented maximal activity against catechol, modest activity against 4-methylcatechol, and minimal activity towards 3-methylcatechol, while C23O2 demonstrated higher activity toward 4-methylcatechol, 3-methylcatechol, 4-chlorocatechol, with activities of 280.69%, 239.20% and 226.39% relative to catechol, respectively. The results revealed that 4-methylcatechol was the best substituted substrate for both C23O1 and C23O2.

## Discussion

Salt-affected soils represent about 40% of the world’s lands^8^, many of which are particularly susceptible to petroleum contamination, posing an environmental problem which cannot be ignored. In the bioremediation of these polluted saline environments, it is necessary to use the corresponding halophilic microorganisms, owing to their dual characteristics of being halophilic and degrading contaminants^31^. From the practical perspective, the halophilic consortium of this study, which had a promising degrading efficiency while using pollutants as the sole carbon and energy source, is well suited for bioremedation application in oil contaminated saline soils. Much effort should also be focused on basic researches to understand the metabolic mechanism and to identify the role of enzymes involved in the degradation metabolism by halophiles. As reported in previous studies, phenanthrene was firstly transformed to cis-dihydrodiol by initial PAH dioxygenase; dihydrodiol dehydrogenase converted dihydrodiol to catechol; and catechol was degraded into aldehydes or acids by C12O or C23O^23^,^32^. We made an enzyme assay of crude cell extracts from the halophiles showing the dynamic change of C23O activity, which mainly played a part in the intermediates metabolism during phenanthrene degradation, and a same phenomenon could be found in Zhao’s report^33^. Two C23O genes were obtained through clone library screening, and the gene relative expression levels were determined by RT-PCR assay. The results showed the newly found C23O genes were expressed during degradation, and this demonstrated that the two C23Os had participated in the phenanthrene mineralization process. Overall, this work provided a viable model to study the role of enzymes in aromatic hydrocarbons degradation by a halophilic consortium and to extract some functional enzymes from this consortium.

As environmental friendly, economical, and clean catalysts, enzymes are enjoying increasing popularity in the chemical industry and envitonmental remediation. Moreover, some halostable enzymes, such as cellulase^34^, endoglucanase^35^, proteases^36^,^37^ and esterase^38^ with their special characterizations under saline conditions, are showing their potential commercial value in various fields. In this case, it is also necessary to study some halophilic or halotolerant enzymes catalyzing aromatic hydrocarbon degredation in saline environment. C23O is the key enzyme of many bacterial pathways for aromatic compounds degradation and presents distinct physicochemical properties in bacterial strains from different sources. In previous studies^24^, C23Os (EC 1.3.11.2) form a large protein family that is divided into several subgroups. In this study, according to the phylogenetic tree constructed by the C23O amino acid sequences from subfamily I.2.A, two C23Os and their homologues formed a monophyletic group in the tree (group II) and were distinguished from other classic C23Os in group I (Fig. 3), which were classified by Eltis and Bolin in 1996 and no further updated since then. In group II, six relative proteins were from sequenced microbial genomes, and thus none of them was characterized; while the C23O in *Pseudomona*s sp. G67 was just verified its expression activity on the gene level^39^. The two C23Os in our study were characterized and thus could be the representative of this new group. Furthermore, C23Os amino acid sequences (Fig. S3) and compositions (Table 1) were compared to further distinguish the novel enzymes. Despite the consistency of conserved residues, some sites of the two C23Os were obviously changed shown in Fig. S3 and the residues compositions were slightly different, suggesting the two C23Os a new feature. A higher level of structure analysis is essential to illuminate these differences in further study. Overall, these novel C23Os made a supplement to the traditional classification by Eltis and Bolin^24^.

Although many C23Os have been extracted or purified from nonhalophiles^40^,^41^,^42^,^43^, no experiments were performed to examine their properties under saline conditions. We particularly investigated the effects of salts on the novel enzymes in this work. The characterization of C23O1 and C23O2 revealed their strong tolerance to NaCl from 0% to 30% salinity (Fig. 4a). In other reports, some halotolerant enzymes such as an esterase^38^, a serine proteinase^36^ and two textracellular proteases^37^ also presented high activity over a wide range of salinity. Moreover, the stability of C23O1 and C23O2 performed better at high salinity than absence of NaCl (Fig. 4b,c), with a similar phenomenon of the halo-stability of a halostable cellulase in Wang’s report^34^. In addition, unlike most halophlic enzymes which are likely to be inactivated at less than 2 M NaCl or KCl concentration^44^, the two enzymes were still active without NaCl. It is reasonable to propose that the two C23Os were halotolerant enzymes and the same definition could be found in several other studies^34^,^38^. It is a limit that halophilic enzymes easily lose activities at low salinity for that they require high concentration of salt to establish an osmotic balance^44^. Instead, halotolerant enzymes like C23Os in this study could still exhibit activities under low salt environment and show tolerance to certain high salinity, which are of major biotechnological interests from the practical perspective. This result also implied that the enzymes could exhibit a halotolerant feature in hydrocarbons degradation by halophiles in saline environment. So far, it is the first report that C23Os, which were isolated from halophilic origins, displayed activity over a broad range of salinity.

To deeply elucidate the halotolerance property of the two C23Os, composition calculation and electrostatic potential analysis were implemented. Halophilic adaptation was usually characterized by a general increase in the content of acidic residues, Asp and Glu^45^. As shown in Table 1, C23O1 and C23O2 had a mild increase in acidic residue content than classic ones. Moreover, the two C23Os also differed from the halophilic enzymes, which usually had a large excess of acidic amino acids (approximately 4-fold) than non-halophilic enzyme^46^. Thus the slight raise in acidic residues content of C23O1 and C23O2 might contribute to their halotolerant feature. On the other hand, as many studies revealed that the abundance of negative charges on the surface was thought to enable the stabilization of the structure of halophilic proteins in high salt concentrations^47^, the halotorelant C23O1 and C23O2 showed the higher negative electrostatic potential on the surface compared with the classic C23O (Fig. S4). The similiar phenomena were also observed in a halotolerant esterase^38^ and a halotolerant endoglucanase^35^. This further confirmed the haloterance property of the two C23Os from a structure aspect. This result also could be the explanation that the enzyme activity kept longer time at 10% and 20% NaCl concentration than in the absence of NaCl in Fig. 4b,c, when surface acidic residues could bind hydrated ions, leading the proteins to remain soluble and properly folded at high salt concentration^44^,^48^. In addition, although some reports^44^,^46^ had indicated that the halophilic property of enzymes had some relationships with hydrophobic or polar residues content, no consistent conclusion was made. It was also undefined whether the hydrophobic or polar residues changes in Table 1 were correlated with the halotolerant feature. As few reports described the characteristics of amino acids in halotolerant enzyme, this study provided some primary information to this field.

From the application view, we also examined other specific properties of the novel enzymes. The two C23Os had wide suitable temperature ranges, 20–60 °C and 30–80 °C respectively. Several C23Os of non-halophilic origins, such as *Stenotrophomonas maltophilia* strain KB2 (30 °C)^49^ and *Pseudomonas* strain S-47 (30–35 °C)^50^, had been found that their optimal temperatures were relatively low. And to our knowledge, there were only two reports described that the C23Os could exhibit activity at high temperature^51^,^52^. Based on the salt-tolerant and thermotolerant properties, these two enzymes may be suited to particular harsh conditions. On the other hand, metal ions play catalytic roles or purely structural role in many proteins. As with other extradiol dioxygenases which requires ferrous ions as the structural role for their activities^53^, the two C23Os activities in this study were enhanced by approximately 3-fold with the presence of Fe^3+^ or Fe^2+^. In contrary, the other C23Os in Table 2 were not positively affected and even slightly inhibited with Fe^3+^, such as in *Stenotrophomonas maltophilia* KB2. The similiar phenomena could also be observed among the four C23Os with the presence of Cu^2+^ and Al^3+^ in Table 2. In addition, some C23Os have shown to be active with Mn^2+^ or Mg^2^ ^54^, which did not apparently impact the two C23Os in this study. Metal ions usually resulted in conformational changes in protein structure, leading to activity loss^55^, or provided correct catalytic site to support substrate binding, enhancing the activity^56^. In conclusion, almost all metal ions used in this study had no significantly negative effects on the two enzymes, making them very useful in bioremediation with coexistence of metals and aromatic hydrocarbons.

This study further examined substrate specificity of C23O1 and C23O2. Previous studies revealed that C23O could catalyze monocyclic catechols and/or polycyclic ones. As the two C23Os did not oxidize 1, 2-dihydroxynaphthalene, they might belong to monocyclic dioxygenases. The two C23Os could metabolize certain substituted catechols, reflecting their wider substrate specificity. Moveover, C23O1 prefered catechol instead of substituted one as the best substrate, and the similiar decreased activities for substituted catechols were also found in C23O-S and C23O-P47 in Table 3. In contrast, C23O2 showed higher specific activity toward substituted catechols. As the substrate specificity was in connection with protein structure to some extent^57^, the distinct substrate metabolism feature of the two enzymes suggested the two C23Os had different enzyme structure and function. In addition, this substrate specificity test also suggested that the two C23Os in this study played a major role in the downstream pathway of phenanthrene degredation. Particularly, the primers used in this study were designed only for C23Os in subfamily I.2.A, and then two novel C23O genes were found. To get more diverse genes, other primers could be designed and utilized. In this case, further research concerning the characterization of some enzymes in upstream pathway, such as NAH-like dioxygenase, or other enzymes (e.g. C12Os) involved in the catabolism from this halophilic consortium are essential to elucidate the catabolic mechanism in phenanthrene degredation by halophiles.

To sum up, this work cloned two novel C23Os from halophiles for the first time, making a supplement to the phylogeny knowledge of extradiol dioxygenases, and this study also characterized the halotolerant enzymes involved in the aromatic hydrocarbons degradation, showing their specific properties and futher potential to remediate oil pollution in saline environment.

## Materials and Methods

### Chemicals, strains and enzymes

Phenanthrene was purchased from Alfa Aesar (England) and antibiotics used in this study were obtained from Sigma-Aldrich (United States). *E. coli* Top 10 and *E. coli* BL21 (DE3) strains were used for propagation of plasmids and overexpression. Restriction endonucleases, Taq DNA polymerase, DNAiso reagent, RNAiso Plus, DNase I, cDNA Synthesis Kit, SYBR^®^ Premix DimerEraser™ and T4 DNA ligase were obtained from Takara (Takara, Dalian, China) and used according to manufacturer’s instructions. The expression vector pET-28a (+) and pMAL-c5X were purchased from Invitrogen (Shanghai, China) and New England Biolabs (USA) respectively.

### Culture enrichment

For enrichment cultures, soil samples were taken from the chronic oil-contaminated and saline soil of the Shengli Oilfield in Shandong Province, China. Using phenanthrene (100 mg/L) as the sole carbon and energy source, a moderately halophilic bacterial consortium (HF-1) capable of degrading phenanthrene was enriched in darkness at 30 °C and shaking at 150 rpm for 15 days. Culture conditions and sea salt-defined media (SSDM) were prepared as described by Zhao *et al.*^6^.

### Preparation of crude cell extracts and enzyme activity assays

The halophilic bacterial consortium HF-1 was inoculated into SSDM with phenanthrene (100 mg/L). Cells were sampled at the fixed time intervals, harvested by centrifugation (8000 × *g* for 10 min at 4 °C), washed with 50 mM phosphate buffer (pH7.5), and then lysed using ultrasound at 200 W in an ice-bath (3-s period followed by a 2-s interval, 80 times). Cell debris was removed by centrifugation at 20,000 × *g* for 60 min at 4 °C. The supernatant solution was used as crude cell extract for the enzyme assays. C23O activity was measured with standard method by determining the formation of 2-hydroxymuconic semialdehyde spectrophotometrically at 375 nm (λ_375_ = 36,000 M^−1^cm^−1^), in a reaction mixture containing 20 μL of catechol (50 mM), 960 μL of phosphoric buffer pH 7.4 (50 mM) and 20 μL of crude extract^49^. One unit of specific activity was defined as the conversion of 1 μM substrate by 1 mg protein per min at 37 °C. Protein concentration of the crude extract was determined by the Bradford method using bovine serum albumin as a standard.

### C23O gene clone library construction

The C23O genes were amplified using primers at conditions described by Junca and Pieper^58^. To construct a clone library, the PCR products were purified and ligated into PMD-19 T. The resulting plasmids were then transformed into competent high-efficiency *E. coli* Top 10 cells following the supplier’s instructions. One hundred white colonies were picked and sequenced, with two novel C23Os found in the clone library. As genes diversity might be related with sample surroundings or specific enrichment conditions, the obtained C23O genes in this study incidentally turned out to be novel ones in this specific bacterial consortium from the saline environment. The two C23O sequences reported in this study are available from GenBank (accession number: KF261118 and KF261119).

### RNA isolation, preparation of cDNA and Quantitative real-time PCR (Q-PCR) analysis

Q-PCR was used to investigate whether the two C23Os were involved in phenanthrene degradation. The bacterial consortium HF-1 were harvested by centrifugation (3000 × *g* for 10 min at 4 °C), washed with SSDM, kept shaking for two days, harvested again and suspended with SSDM. In subsequent experiments, 200 μL of this cell suspension was inoculated into 100 mL of fresh culture containing 100 mg/L phenanthrene to evaluate the induced expression of the two C23Os and the culture condition was as same as enrichment culture. 2 mL bacterial samples were withdrawn from the culture in triplicate at an interval of 4 h. Total DNA and RNA were extracted directly from each sample using the DNAiso Reagent and RNAiso Plus. Contaminated DNA in the RNA preparation was removed using DNase I according to the manufacturer’s instructions. Reverse transcription (RT) was carried out on total extracted RNA using cDNA Synthesis Kit according to the manufacturer’s protocol.

C23O genes and transcripts were quantified simultaneously by a Q-PCR apparatus (Bio-Rad, America) and followed by a melt curve analysis with the primer pairs: C23O1-5′-AAGTTCTCCGTGGTGCTGC-3′, C23O1-5′-TACCTGCTCAACCTCAACG-3′; and C23O2-5′-AGTTCTCGGTCGTGCTGGT-3′ C23O2-5′-GACCACAGTCCTTCAGCTCAC-3′. The PCR reactions were performed using 30 cycles of 45 s at 96 °C, 30 s at 60 °C and 45 s at 72 °C. Both primer pairs were highly specific, and no aspecific fragments were generated. Standard curves were produced using the plasmid DNA. The two C23O genes relative expression levels were calculated based on the transcript/gene ratio.

### Protein production and purification

Vectors have an effect on protein heterologous expression and to get the suitable soluble expression in *E.coli*, C23O1 and C23O2 were cloned into the expression vector pET28a (+) and pMAL-c5X, respectively. The C23O1 gene was amplified by PCR with the primers pairs which was added a *Nde I* and *Sac I* site (underlined): 5′- GCGCATATGATGAAAAAAGGTG-3′ and 5′-ACTGAGCTCTTAGGTGAGAACGGT-3′, and C23O2 was amplified with the primers pairs which was added a *Nde I* and *EcoR I* site (underlined): 5′-TGAGAGTGCATATGATGAAAAAAGGTG-3′ and 5′-TTGGAATTCTCAGGTCAGCACG-3′. The constructs were transformed into *E.coli* BL21 (DE3) for expression analysis.

Strains BL21 (DE3)-pET28a(+)-C23O1 or BL21 (DE3)-pMAL-c5X-C23O2 were grown in Luria-Bertani containing suitable antibiotics to an OD_600_ of 0.6, at which 0.5 mM isopropyl-β-D-thiogalactopyranoside (IPTG) was added to induce the protein expression. After 5 h, cells were centrifuged, washed and then disrupted by sonication. Cell debris was removed by centrifugation at 12,000 rpm for 30 min at 4 °C and the supernatant solution was used to purify proteins. C23O1 was immobilized onto a column packed with Ni^2+^-nitrilotriacetic acid (Novagen, Germany) and digested by thrombin, while C23O2 was purified by amylose affinity chromatography according to manufacturer’s instructions and digested with Factor Xa (New England Biolabs). Finally, the protein purification process was analyzed by sodium dodecyl sulfate-polyacrylamide gel electrophoresis (SDS-PAGE) on 12% polyacrylamide mini gels. The gels were taken photos with a camera and the images were processed to be patched together by Photoshop. The molecular mass of protein was determined by comparing with standard protein.

### C23O phylogenetic tree construction

The BLAST program (http://www.ncbi.nlm.nih.gov/BLAST/) was used for gene homology searches. Phylogenetic trees were constructed using the neighbour-joining methods with MEGA version 5.0 and the evolutionary distance was calculated by p-distance model. The C23Os sequences were selected from the GenBank database with the principles: some ones were with the highest similarities after BLAST and others were previously reported C23Os from non-halophiles (classic C23Os) classified by Eltis and Bolin^24^. In addition, a corresponding maximum-parsimony tree was also constructed using MEGA version 5.0. The bootstrap analyses were all based on 1000 replications.

### C23O amino acids sequence analysis

The sequences alignment was performed by Clustal W (see Fig. S3). Aligned sequences are selected from classic C23Os in the constructed phylogenetic tree in Fig. 3. Some structure elements knowledge (alfa-helix, beta-strand, substrate-binding-site and inner or surface position) referred to C23O of *Pseudomonas putida* mt2 in the Brookhaven Protein Data Bank with accession code 1mpy; while the messages of Fe-binding-site, conserved active residues and residues forming the active site pocket were based on the reported three-dimensional structure of C23O in *Pseudomonas putida* mt2^29^.

### C23O electrostatic potential analysis

Three-dimensional (3D) structures of C23O1 and C23O2 were modeled based on the structure of C23O from *Pseudomonas putida* mt2 (PDB no. 1MPY). The surface electrostatic potential of the enzymes was calculated by Discovery Studio 2.5 software (Accelrys, SanDiego, CA, USA).

### Effects of salinity and metal ions on C23Os activity

Salt influence on enzyme activity was examined in 50 mM phosphate buffer (pH 7.4) that contained various concentrations of NaCl (0–30%, w/v). The values were percentages of enzyme activity at 0% salinity. To determine the halo-stability of C23Os, 0%, 10% and 20% salinity were selected for this test. The C23Os were pre-incubated in buffers with the specified NaCl concertrations at 4 °C for the designated periods of time (see Fig. 4b for stability examination; 30 min for activity test), and the residual activities were measured by the standard method mentioned above. Effects of various metal ions were investigated by adding these substances into the C23O solution at a final concentration of 5 mM. Then the mixtures were pre-incubated at 4 °C for 30 min, and measured by standard enzyme assay method, as described above. The activity assayed in the absence of metal ions was recorded as 100%. Each value was an average from triplicate tests.

### Determination of optimal temperature and substrate specificity of C23Os

To determine the optimal temperature, enzyme assays were conducted in a temperature range of 0–90 °C. At each temperature, the enzyme and substrate solutions were first pre-incubated to the target temperature individually, and then mixed to initiate the enzymatic reaction. Thermostability of the enzymes was also measured using the incubator from 0 °C to 50 °C for 24 h and the residual enzyme activity was recorded at 6 h intervals during incubation period. The enzyme activity was determined by the standard method. The substrate specificity of both C23Os was examined with 3-methylcatechol, 4-methylcatechol, 4-chlorocatechol, and 1, 2-dihydroxynaphthalene (1, 2-DHN) using the spectrophotometric method^59^. Each value was an average from triplicate tests.

## Additional Information

**How to cite this article**: Guo, G. *et al.* Isolation and characterization of two novel halotolerant Catechol 2, 3-dioxygenases from a halophilic bacterial consortium. *Sci. Rep.* **5**, 17603; doi: 10.1038/srep17603 (2015).

## Supplementary Material

Supplementary Information

## Figures and Tables

**Table 1:** C23Os amino acids composition calculation and comparison.

**Enzymes**	**Positive**^c^	**negative**	**hydrophobic**	**polar**	**Total number**
*Pseudomonas putida* PS35X (CAA54847)^a^	15.635	15.309	50.814	18.241	307
Plasmid pVI150 (AAA27512)	15.309	14.984	51.140	18.567	307
*Pseudomonas putida* H (CAA56747)	15.309	15.309	51.140	18.241	307
Plasmid pDK1 (AAA23353)	14.984	15.961	51.140	17.915	307
Plasmid NAH7 (AAA98183)	14.984	15.635	52.443	16.938	307
*Pseudomonas* sp. IC (AAA20982)	15.961	15.309	51.140	17.590	307
*Pseudomonas aeruginosa* JI104 (CAA43145)	16.287	15.309	50.489	17.915	307
*Bacillus subtilis* (CAA01203)	16.013	15.686	50.000	18.301	306
*Pseudomonas putida* mt2 (CAA24490)	15.635	15.635	50.489	18.241	307
Average value^b^	15.569 ± 0.460	15.460 ± 0.292	50.977 ± 0.681	17.994 ± 0.487	
C23O1 (AGW45615)	15.635	16.938	50.814	16.612	307
C23O2 (AGW45616)	15.309	16.287	52.443	15.961	307

^a^The first nine sequences are from the classic C23Os in Fig. 3.

^b^The values in this line are the averages of relevant residues content in the nine classic C23Os sequences.

^c^The values in this column are the ratio of the number of positive residues to total amino acids number and values in other column are calculated like this way.

**Table 2:** Comparison of metal ions effects on different C23Os^a^.

Metal ions	C23O1	C23O2	C23O-S^b^	C23O-G^c^
Control	100	100	100	100
Mg^2+^	88.1	100.1	–	120
K^+^	81.2	95.8	–	74
Mn^2+^	98.2	101.8	46.0	230
Cu^2+^	218.2	256.7	0.5	76
Fe^3+^	246.8	377.4	67.2	123
Fe^2+^	265.6	365.8	72.3	–
Al^3+^	120.9	165.6	84.5	–

^a^Expressed as a percentage of the C23Os specific activity toward catechol without metal ions which is set as 100%. Data are expressed as averages from triplicate experiments.

^b^C23O-S refers to the C23O from *Stenotrophomonas maltophilia* KB2^43^.

^c^C23O-G represents the C23O from *Gordonia polyisoprenivorans*^60^.

**Table 3:** Comparison of substrate specificity among different C23Os^a^.

Substrate	C23O1	C23O2	C23O-P5^b^	C23O-S^c^	C23O-P47^d^
catechol	100	100	100	100	100
3-methcatechol	49.5	239.2	13.7	10.6	33.7
4-methcatechol	87.4	280.7	106.3	82.3	62.8
4-chlorocatechol	50.8	226.4	203.8	0	71.4
1, 2-dihydroxynaphthalene	0	0	—	—	—

^a^Expressed as percentage of specific activity with catechol which is set as 100%. Data are expressed as averages from triplicate experiments.

^b^C23O-P5 stands for the C23O from *Planococcus* sp.S5^42^.

^c^C23O-S refers to the C23O from *Stenotrophomonas maltophilia* KB2^43^.

^d^C23O-P47 represents the C23O from *Pseudomonas* sp. S-47^48^.
