# Supplementary material for: Isolation and characterization of two novel halotolerant Catechol 2, 3-dioxygenases from a halophilic bacterial consortium
Source: Sci Rep. 2015 Dec 1;5:17603. doi: 10.1038/srep17603 (PMC4664950; doi:10.1038/srep17603)
Supplement: Supplementary Information [file srep17603-s1.pdf]

Isolation and characterization of two novel halotolerant Catechol 2, 3-dioxygenases from a halophilic bacterial consortium

Authors:

Guang Guo, Tingting Fang, Chongyang Wang, Fang Tian, Yong Huang, Qijia Cui, Hui Wang *

School of Environment, Tsinghua University, Beijing, 100084, China

Address correspondence to Hui Wang: wanghui@tsinghua.edu.cn.

* Present address: School of Environment, Tsinghua University, Beijing 100084, China

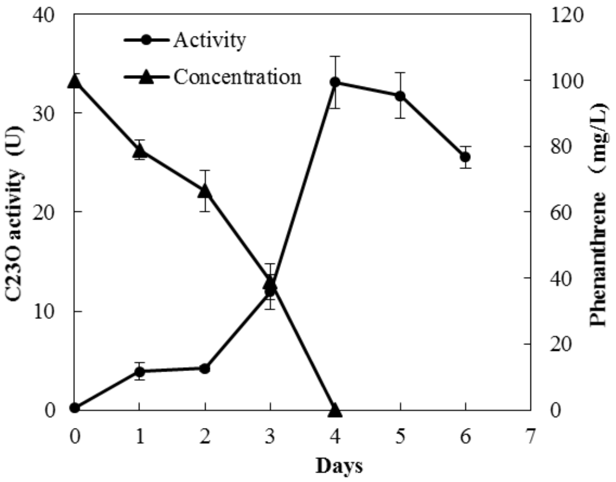

Supplementary Figure S1 C23O activity during phenanthrene degradation by the halophilic bacterial consortium at an initial concentration of 100 mg/L

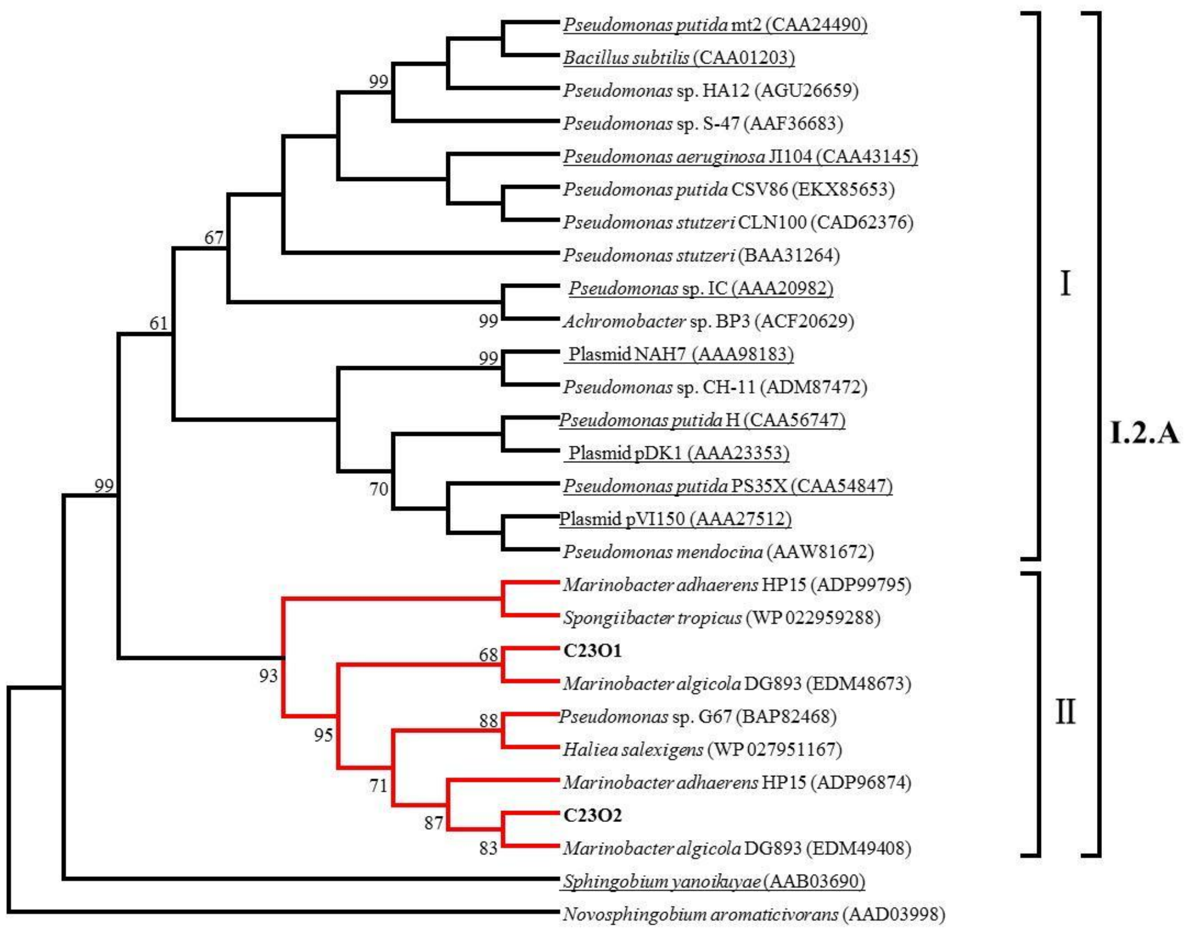

Supplementary Figure S2 Phylogenetic maximum-parsimony tree based on the C23O amino acid sequences. The clones isolated in this study are indicated with boldface type. The sequences with the underline are classic C23Os which were classified by Eltis and Bolin. The C23O amino acid sequences of *Sphingobium yanoikuyae* (AAB03690) and *Novosphingobium aromaticivorans* (AAD03998), two members of subfamily I.2.B were used as an outgroup. Bootstrap values (>60) expressed as percentages of 1000 replications are indicated at the branch points.

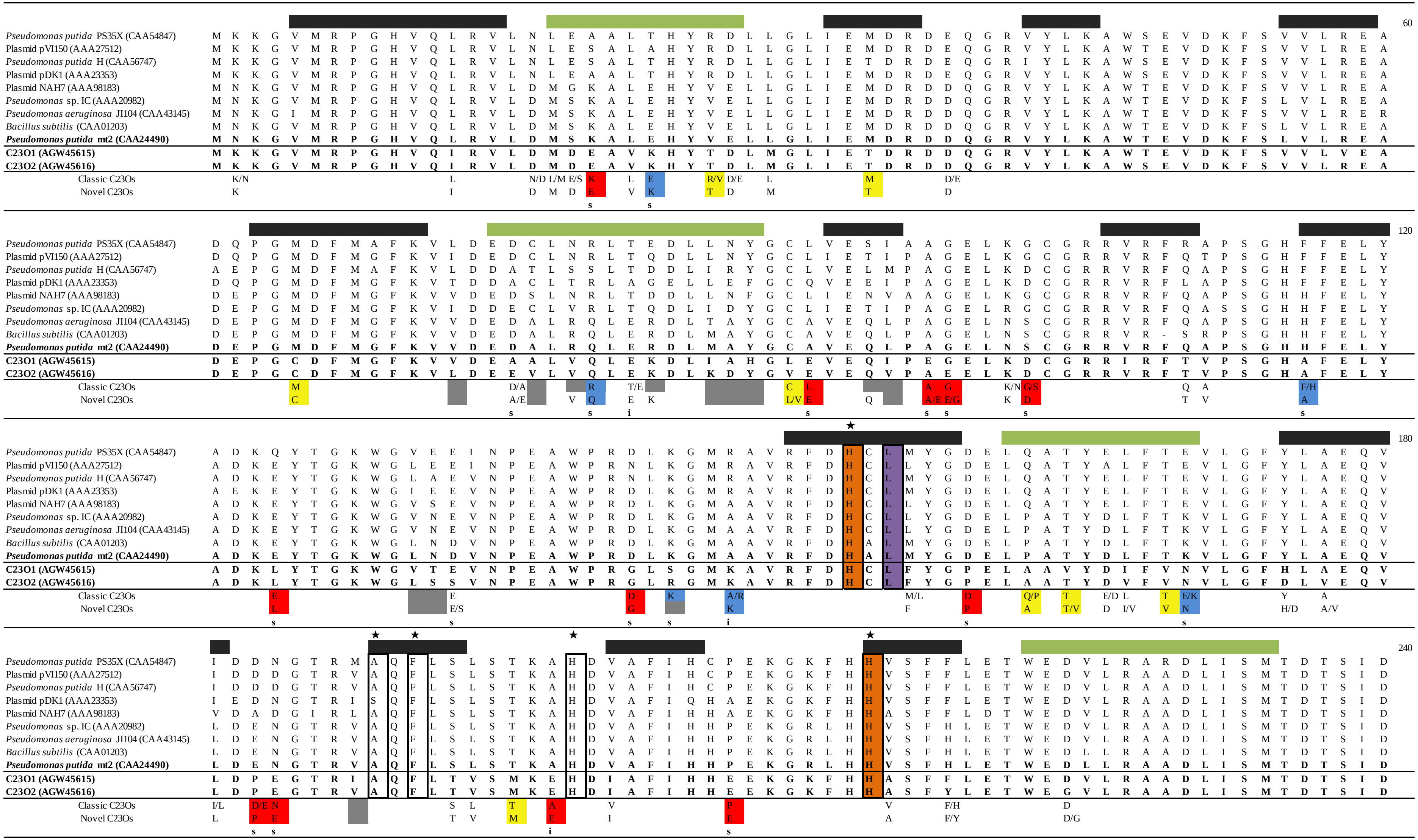
60
120
180
240
Pseudomonas putida PS35X (CAA54847)
Plasmid pVI150 (AAA27512)
Pseudomonas putida H (CAA56747)
Plasmid pDK1 (AAA23353)
Plasmid NAH7 (AAA98183)
Pseudomonas sp. IC (AAA20982)
Pseudomonas aeruginosa JI104 (CAA43145)
Bacillus subtilis (CAA01203)
Pseudomonas putida mt2 (CAA24490)
C23O1 (AGW45615)
C23O2 (AGW45616)
Classic C23Os
Novel C23Os

300

						★									★										★																																			
Pseudomonas putida PS35X (CAA54847)	I	G	P	T	R	H	G	L	T	H	G	K	T	I	Y	F	F	D	P	S	G	N	R	N	E	V	F	C	G	G	D	Y	N	Y	Q	D	Q	K	P	V	T	W	L	A	K	D	L	G	K	A	I	F	Y	H	D	R	V	L	N	E
Plasmid pVI150 (AAA27512)	I	G	P	T	R	H	G	L	T	H	G	K	T	I	Y	F	F	D	P	S	G	N	R	N	E	V	F	C	G	G	D	Y	N	Y	Q	D	H	K	P	V	T	W	L	A	K	D	L	G	K	A	I	F	Y	H	D	R	V	L	N	E
Pseudomonas putida H (CAA56747)	I	G	P	T	R	H	G	L	T	H	G	K	T	I	Y	F	F	D	P	S	G	N	R	N	E	V	F	C	G	G	D	Y	N	Y	Q	D	H	K	P	V	T	W	L	A	K	D	L	G	K	A	I	F	Y	H	D	R	V	L	N	E
Plasmid pDK1 (AAA23353)	I	G	P	T	R	H	G	L	T	H	G	K	T	I	Y	F	F	D	P	S	G	N	R	N	E	V	F	C	G	G	D	Y	N	Y	P	D	H	K	P	V	T	W	T	A	D	Q	L	G	K	A	I	F	Y	H	D	R	I	L	N	E
Plasmid NAH7 (AAA98183)	I	G	P	T	R	H	G	L	T	Q	G	K	T	I	Y	F	F	D	P	S	G	N	R	C	E	V	F	C	G	G	N	Y	N	Y	P	D	H	K	P	V	T	W	L	A	K	D	V	G	K	A	I	F	Y	H	D	R	V	L	N	E
Pseudomonas sp. IC (AAA20982)	I	G	P	T	R	H	G	L	T	H	G	K	T	I	Y	F	F	D	P	S	G	N	R	S	E	V	F	C	G	G	N	Y	S	Y	P	D	H	K	P	V	T	W	L	A	K	D	L	G	K	A	I	F	Y	H	D	R	V	L	N	E
Pseudomonas aeruginosa JI104 (CAA43145)	I	G	P	T	R	H	G	L	T	H	G	K	T	I	Y	F	F	D	P	S	G	N	R	S	E	V	F	C	G	G	N	Y	S	Y	P	D	H	K	P	V	T	W	L	A	K	D	L	G	K	A	I	F	Y	H	D	R	V	L	N	E
Bacillus subtilis (CAA01203)	I	G	P	T	R	H	G	L	T	H	G	K	T	I	Y	F	F	D	P	S	G	N	R	N	E	V	F	C	G	G	D	Y	N	Y	P	D	H	K	P	V	T	W	T	T	D	Q	L	G	K	A	F	F	Y	H	D	R	I	L	N	E
***Pseudomonas putida* mt2 (CAA24490)**	**I**	**G**	**P**	**T**	**R**	**H**	**G**	**L**	**T**	**H**	**G**	**K**	**T**	**I**	**Y**	**F**	**F**	**D**	**P**	**S**	**G**	**N**	**R**	**N**	**E**	**V**	**F**	**C**	**G**	**G**	**D**	**Y**	**N**	**Y**	**P**	**D**	**H**	**K**	**P**	**V**	**T**	**W**	**T**	**T**	**D**	**Q**	**L**	**G**	**K**	**A**	**I**	**F**	**Y**	**H**	**D**	**R**	**I**	**L**	**N**	**E**
**C23O1 (AGW45615)**	**I**	**G**	**P**	**T**	**R**	**H**	**G**	**L**	**T**	**H**	**G**	**K**	**T**	**I**	**Y**	**F**	**F**	**D**	**P**	**S**	**G**	**N**	**R**	**N**	**E**	**V**	**F**	**C**	**G**	**G**	**D**	**Y**	**T**	**Y**	**P**	**D**	**H**	**K**	**P**	**V**	**T**	**W**	**Q**	**A**	**E**	**Q**	**L**	**G**	**K**	**A**	**I**	**F**	**Y**	**H**	**D**	**R**	**V**	**L**	**N**	**E**
**C23O2 (AGW45616)**	**I**	**G**	**P**	**T**	**R**	**H**	**G**	**L**	**T**	**H**	**G**	**T**	**T**	**I**	**Y**	**F**	**F**	**D**	**P**	**S**	**G**	**N**	**R**	**N**	**E**	**V**	**F**	**C**	**G**	**G**	**D**	**Y**	**F**	**Y**	**P**	**D**	**H**	**K**	**P**	**V**	**T**	**W**	**K**	**T**	**E**	**E**	**L**	**G**	**K**	**A**	**I**	**F**	**Y**	**H**	**D**	**R**	**V**	**L**	**N**	**E**
Classic C23Os												K																					N/S										L/T		K/E	D/Q														
Novel C23Os												K/T																					T/F										K/Q		E	Q/E														
																																											s		s															

Pseudomonas putida PS35X (CAA54847)	R	F	L	T	V	L	T
Plasmid pVI150 (AAA27512)	R	F	L	T	V	L	T
Pseudomonas putida H (CAA56747)	R	F	M	T	V	L	T
Plasmid pDK1 (AAA23353)	R	F	M	T	V	L	T
Plasmid NAH7 (AAA98183)	R	F	M	T	V	M	T
Pseudomonas sp. IC (AAA20982)	R	F	M	T	V	L	T
Pseudomonas aeruginosa JI104 (CAA43145)	R	F	M	T	V	L	T
Bacillus subtilis (CAA01203)	R	F	M	T	V	L	T
***Pseudomonas putida* mt2 (CAA24490)**	**R**	**F**	**M**	**T**	**V**	**L**	**T**
**C23O1 (AGW45615)**	**R**	**F**	**L**	**T**	**V**	**L**	**T**
**C23O2 (AGW45616)**	**R**	**F**	**M**	**T**	**V**	**L**	**T**
Classic C23Os							
Novel C23Os							

Legend of structure elements :

- Alfa-helix
- Beta-strand
- Substrate-binding-site
- Fe-binding-site
- ★ Conserved active site
- Residues forming the inner channel wall of the active site pocket

Legend of residue changes:

- Positive residues conversion
- Negative residues conversion
- Hydrophobic / polar residues conversion
- irregular conversion
- i Inner charged residues
- s Surface charged residues

Supplementary Figure S3 Multiple sequence alignment of C23O amino acid sequences. Aligned sequences are from C23Os according to the constructed phylogenetic tree in figure 3. The first nine sequences are the classic C23Os. The information about the sequences structure elements is presented with special forms referred to the legend at lower right corner. Residue conversions between the two types are summarized in the bottom lines and some obvious residue changes are highlighted with different back colors according to legend at lower right corner.

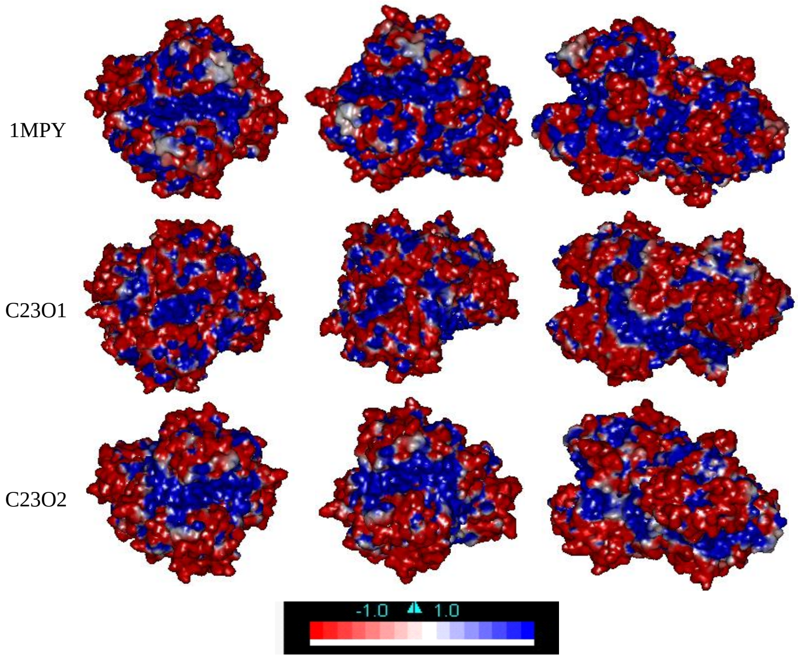

Supplementary Figure S4 Surface electrostatic potentials of C23O1, C23O2 and C23O from *Pseudomonas putida* mt2 (PDB no. 1MPY) obtained using Discovery Studio 2.5 software. The first, second and third column were seen from the right, top and front of the structures, respectively, with the red color corresponding to negatively charged residues and the blue surface corresponding to positively charged residues (color figure line).

Supplementary Table S1 Sequence similarity between selected C23O reference sequence [a] and C23O1 or C23O2

**Reference sequence**	**C23O1**	**C23O2**
**Group 1**		
Pseudomonas putida mt2 (CAA24490)	79%	79%
Pseudomonas sp. HA12 (AGU26659)	79%	79%
Bacillus subtilis (CAA01203)	79%	79%
Pseudomonas sp. S-47 (AAF36683)	79%	79%
Pseudomonas stutzeri (BAA31264)	81%	79%
Pseudomonas stutzeri CLN100 (CAD62376)	80%	79%
Pseudomonas aeruginosa JI104 (CAA43145)	80%	79%
Pseudomonas putida CSV86 (EKX85653)	80%	79%
Pseudomonas sp. IC (AAA20982)	78%	78%
Achromobacter sp. BP3 (ACF20629)	79%	79%
Plasmid NAH7 (AAA98183)	78%	77%
Pseudomonas sp. CH-11 (ADM87472)	77%	76%
Plasmid pDK1 (AAA23353)	78%	76%
Pseudomonas putida H (CAA56747)	78%	79%
Pseudomonas putida PS35X (CAA54847)	77%	76%
Plasmid pVI150 (AAA27512)	78%	77%
Pseudomonas mendocina (AAW81672)	79%	77%
**Group 2**		
Spongiibacter tropicus (WP 022959288)	83%	81%
Marinobacter adhaerens HP15 (ADP99795)	90%	87%
Marinobacter algicola DG893 (EDM48673)	**93%**	90%
Pseudomonas sp. G67 (BAP82468)	91%	92%
Haliea salexigens (WP 027951167)	91%	92%
Marinobacter adhaerens HP15 (ADP96874)	87%	91%
Marinobacter algicola DG893 (EDM49408)	88%	**95%**

[a] These reference sequences corresponded to the ones in Figure 3.
